# Documenting the Impact of Infra Low Frequency Neurofeedback on Underserved Populations With Complex Clinical Presentations

**DOI:** 10.3389/fnhum.2022.921491

**Published:** 2022-05-26

**Authors:** Matthew J. Fleischman

**Affiliations:** Neurofeedback Advocacy Project, Eugene, OR, United States

**Keywords:** ILF neurofeedback, neurofeedback, wicked problems, infra low frequency neurofeedback, patient-reported outcome (PROMs), treatment outcomes

## Abstract

There is a crisis in mental health. The demand for services is huge; the efficacy of current services is lacking, and the traditional path to developing effective treatments is not working. This paper describes an approach based on implementing infra low frequency (ILF) neurofeedback in agencies that work with the underserved and collecting data on client’s concerns, behavior, quality of life and cognitive performance. We also track session-by-session changes in client concerns and their relation to ILF neurofeedback protocols. Results are reviewed for over 300 clients who have been seen in such agencies. Limitations and future directions are discussed for this model.

## Introduction

### Kind and Wicked Problems

There is little dispute that we face a crisis in mental health and that the current mental health system is not serving the millions who desperately need help, a conclusion drawn by the recent former director of the National Institute of Mental Health (Insel, 2022). It is unclear if we have even made much progress in treating mental health problems at all. For example, a review of 435 random controlled trials (RCT) of psychotherapies for children showed no improvement in outcomes in the last 53 years (Weisz et al., 2019). In response, the authors’ recommendation was to “intensify the search for mechanisms of change.” This calls to mind the statement attributed to Einstein, “The definition of insanity is doing the same thing over and over and expecting a different result.” And if we are looking to achieve a different result, we should consider the difference between a Kind (sometimes referred to as a Tame Problem) and a Wicked problem, a distinction formally described 50 years ago (Rittel and Webber, 1973).

A Kind problem is not necessarily an easy problem. Rather it is a problem that theoretically can be solved. Kind problems are typically solved using the scientific method: forming a hypothesis, defining terms, making a prediction, running a controlled test, and refining the hypothesis based on the results. A proof that the solution is correct is that it is replicable.

In contrast, a Wicked problem is a problem that because of incomplete, contradictory, and changing requirements that are often difficult to recognize, is difficult or impossible to solve. Typically, a Wicked problem involves many stakeholders with different values and priorities. The roots of the problem are complex and tangled. Every Wicked problem can be considered to be a symptom of another problem. Wicked problems are difficult to come to grips with and the solution changes with every attempt to address it. To put matters in a current context, developing a vaccine for COVID-19 is a Kind problem; distributing the vaccine and getting people to take it is a Wicked one.

By their nature, Wicked problems cannot be solved by searching for the mechanism of change because there is no mechanism of change. RCT can advance our knowledge of mental health disorders. They will not solve the problem of a broken mental health care system, can make us too dependent on the past results of RCTs and fail to appreciate the value of systematic analysis and feedback on treatment and outcomes in real-time. Perhaps worse, the current paradigm retards the adoption of novel treatments that are difficult to package into the constraints of a RCT.

This is certainly the case with neurofeedback, for which to date there are nearly 2,400 published studies conducted over 40 years, including several with formal experimental designs, including animal studies, reversal designs, and comparisons against best practice. Yet neurofeedback is still often disparaged as “investigational.” Clearly some of that resistance by the medical, scientific and insurance industry gatekeepers is probably grounded in unreasonable professional caution or financial self-interest. Another source of resistance is that neurofeedback is a novel treatment paradigm and does not fit into either of the prevailing models of mental health disorders. Namely that they stem from an imbalance or disturbance in brain chemistry or are the consequence of early experiences that must be cognitively or behaviorally unlearned.

### Background on the Neurofeedback Advocacy Project

At this point, it makes sense to introduce myself. I am a licensed psychologist in Eugene, Oregon. I was trained in social learning theory, a model that emphasizes environmental influences and especially parental practices in the treatment of children with behavior disorders. In 1985 I was introduced to neurofeedback by Eugene Peniston, Joel Lubar and Siegfried Othmer, and began integrating it into my professional practice. Over time, I became convinced that neurofeedback was a powerful tool in therapy for helping clients of all types and with all sorts of concerns including emotional, behavioral, and even physical/medical ones. I emphasize that neurofeedback is a tool in psychotherapy because it was always embedded in a therapeutic relationship and always done in conjunction with other modalities including cognitive behavioral therapy, solution-focused approaches, psychoeducation, and family behavior therapy as made best sense with a particular client. I also concluded that neurofeedback is safe. I have used several neurofeedback modalities including quantitative electroencephalogram-based protocols (qEEG), the low energy neurofeedback system (LENS), and frequency-band training. I settled on ILF Neurofeedback because of its effectiveness with clients whose history included significant trauma. However, what convinced me that ILF neurofeedback was “ready for prime time” were two events outside my practice.

I learned that a local non-profit counseling agency introduced neurofeedback as part of psychotherapy and that the program grew to ten therapists and a constant waiting list of over 200 clients. Around the same time another therapist at the local county behavioral health department was permitted to bring in her own neurofeedback equipment to use with her very difficult clients. These were clients with persistent and significant mental illness for whom the courts mandated therapy in lieu of prison. Soon other therapists were noticing the clients she saw were also beginning to talk in group therapy, showed a brighter affect and perhaps most importantly, were showing up for more of their appointments. In fact, her No Show/Late Cancelation rate was only 4% compared to the usual rate of 28%.

Impressed by this anecdotal evidence from agencies that worked with the underserved, I made an offer to three local agencies: I would lend them my neurofeedback equipment if they would send their staff and pay for the training. One agency provided transitional support for felons released from prison; another served forensic clients at the county behavioral health department; and the third was a community mental health agency in an economically hard-pressed community. I also created a method for tracking the results and for gathering some statistics on their client population.

As expected, these agencies all worked with challenging clients. Their average Adverse Childhood Events score of nearly 6, a number that places these clients at high risk for serious mental and physical disorders. Nearly all had multiple co-occurring disorders such as sleep, attention, depression, anxiety and/or pain and all had multiple life stressors. And yet after just 1 week of training plus bi-weekly case conferences, these agency clinicians achieved nearly a 40% reduction in the severity of client-selected, client-rated concerns for the 100 plus clients they saw. Perhaps more impressive, neurofeedback seemed to be highly attractive to the clients. They recommended it to their family and friends, and they showed up for appointments.

The success of this pilot led to the formation of the Neurofeedback Advocacy Project. To that end we developed a model of online training and clinical support to ensure clinical quality and client safety and an online, HIPAA-compliant Results Tracking System (RTS). Over time we have enrolled over 20 sites and have a growing set of outcomes for over 300 clients as of today, all of which was done in the face of COVID restrictions that significantly slowed agency service delivery. And we approached our mission recognizing it as a Wicked problem. Here are the guiding principles we are using:

1. Work in the Real World. From the beginning, if you are researching solutions to real problems, do so in the real world. Implement trials in as many suitable locations as possible, as we recognize the need for diversity, equity, and inclusion in our efforts. This requires going beyond fee-for-service private practice. This is why the Neurofeedback Advocacy Project primarily works with agencies and organizations that work with underserved or difficult-to-engage clients, using their existing staff as the therapists.
2. Move quickly. Test the approach under as many conditions as possible. Use what you learn in each trial to refine what you are doing. Take setbacks as learning experiences rather than a rejection of hypotheses. Revise and try again. While we have had no failures and the clinical results look good across sites, we have revised our training, and also the type of agencies that we have brought into the project.
3. Make sure goals and objectives align among all the concerned parties or establish multiple goals and be prepared to modify goals based on results. We do not simply sign up interested parties. We require a supervisor to participate in the training and offer training to management without expectation that they will ever use it with clients. We have a list of requirements agency heads must agree to in order to join the project, and we work to make the program align with their needs.
4. Make sure goals can be stated in measurable terms. If you cannot “say it with numbers,” what are you actually saying? While our Results Tracking System (RTS)^1^ does ask narrative questions, most of the outcome measures are numeric. We have even developed an Efficacy Score which is the percentage reduction in severity of the client’s concerns divided by the number of sessions. This may be unique as a single metric of client improvement that can be used across all treatments and client populations.
5. Create an efficient mechanism for collecting measurements. If it is too difficult, too costly or you lose too much information, the process will not work. The RTS is built on Google Sheets and by tying it into the clinical intake, therapists and clients appreciate the benefit to them of entering their data and seeing their progress.
6. Emphasize immediacy of data collection and feedback. The fresher the information, the more useful. You cannot correct what you are doing if you only hear about the problem months or years later. Data is live and results are posted to our website^2^ immediately. Because it is stored as a MySQL database, researchers can easily perform all sorts of analyses. As the data set grows, it will be amenable to Big Data studies. And the RTS is multi-lingual in over 80 languages.
7. Minimize barriers to implementing and testing solutions. Keep costs low for both the innovator and the implementer. Look for ways to keep the process self-sustaining. While any project has expenses, using the tools of the Internet such as Zoom and Google Docs lowers the cost of communication and implementation and permits what has evolved from three sites in Oregon to multi-state and now a multinational project. We currently seek organizations and donors to subsidize training to eliminate financial barriers for the agencies.
8. Encourage flexibility. Allow the implementers to adjust the innovation to fit their needs. Nearly every agency has implemented neurofeedback slightly differently in terms of how they select clients, which staff got trained, and how they cover the services.
9. Be open with your results. Do not cherry pick your results or withhold your findings to the end. As noted, our results are updated to the Internet daily.
10. Do not over-claim, but do not minimize the significance of what you do achieve.

## Results

In designing the RTS, we needed a data collection system that was standardized across settings and ensured that data could be aggregated with integrity. It needed to capture client details, treatment details and outcomes in one place, allowing us to analyze outcomes, and in future apply AI to identify patterns that may optimize future approaches. In deciding what metrics to include, first and foremost, the metric needed to have strong face validity. Questionnaires may be meaningful to researchers, but most decision makers are not impressed by a 10-point reduction on a 90 item symptom checklist. Second, because agency clinicians are already burdened with paperwork, anything we did ask had to be as painless as possible and, if possible, be of immediate value to the therapist and client. Third, the measures had to cover a range of outcomes including ones relevant to the client’s concerns, as well as measures of impact on their behavior and quality of life. Subjective ratings and comments are also important as they convey information that cannot always be captured in a numerical rating. We needed measures of healthcare utilization as we suspected that neurofeedback had a positive—though often overlooked—impact. Finally, we wanted to track premature termination and No shows/Late cancelation rates, as these are markers of program effectiveness and acceptance. We built this program using Google Sheets because everyone would have access to it and because, if properly configured, it can be HIPAA compliant.

What follows is a discussion of all the measures and the results to date. Client demographic information is provided in Supplementary Figure 1. Because all data is pushed to our website daily, these numbers and charts are a snapshot in time of our results.

### Reduction in the Severity of Client-Selected, Client-Rated Concerns

The standard treatment research design asks, what is the effect of X treatment on clients with Y problem? This leads to ensuring that clients meet diagnostic criteria as set forth in the DSM and then measuring change using various questionnaires that rate improvement of a symptom or cluster of symptoms. However, in what might be described as an on-going series of ever-changing field studies with varying settings and populations, what disorders are we treating and how do we measure improvement? Unlike a research study that recruits subjects for a specific disorder like PTSD or depression, where we implement neurofeedback, people do not generally seek help for something listed in the DSM. Rather, they seek help for a variety of concerns that may or not map to the DSM. In many cases, people are sent for help not because they believe they have anything wrong with them but at the behest (or demand) of someone else who feels they have something that needs treatment.

In the case of many people who end up being seen in some sort of community mental health setting these clients do not have a single problem like depression or anxiety; they have multiple concerns that map over many diagnoses. Indeed, simply asking the therapist to mark off all the areas such as depression, anxiety, sleep, pain, focus, and attention found the average number of such co-occurring problem areas to be 5. One solution is to list each of these problems as a co-occurring disorder, leaving the person burdened with a dismayingly formal list of what is wrong with them. I would argue that this psychiatric medicalization of suffering is unhelpful to the client; it deprives them of agency and forces the range of human concerns into narrow boxes. For the researcher, it complicates measuring treatment impact because it requires multiple outcome measures, each matched to a diagnosis. This may work in a limited research study, but it does not work in the real world where neither the therapist nor the client has any investment in filling out paperwork. Clearly, we needed a better system for measuring change that would meet several criteria.

First it needed to be applicable to the wide range of presenting concerns as we were implementing neurofeedback in a wide range of agencies serving a wide range of clients. Second, many of the clinicians in these agencies, while licensed, were not qualified to diagnose. Third, the system needed to be very low to no cost to implement. Fourth, it needed to be easy to use and not too time consuming or it would not be used. Fifth, and most important, it should provide a single metric of change across all sites, all clients, and all concerns. To meet all these criteria, we developed the following metric: the reduction in the severity of client-selected, client-rated concerns.

**FIGURE 1:**
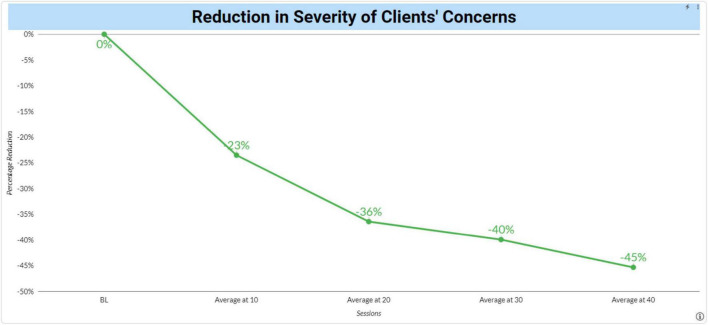
Reduction in severity of clients’ concerns following neurofeedback. Changes from baseline in the severity of clients’ mental health concerns were plotted over time following neurofeedback sessions.

To create this metric, each client is asked, prior to starting neurofeedback, to identify five to eight concerns that they can observe change on a day-by-day basis. To help develop a comprehensive list, they are given a list of possible concerns in all areas including sleep, attention and learning problems, sensory problems, behavioral problems, emotional problems, physical problems, and pain. They are asked to phrase each concern in their own words such that something like “depression” would be converted into what they might notice being able to do if they were not depressed. Once the concerns have been selected, they are asked to rate each concern on a 0–10-point scale for a “Bad Week,” “Good Week” and a “Usual Week” where lower numbers indicate the concern is less severe. Ratings are made by marking a checkbox on a computer screen or tablet. All previous ratings appear on the screen so that ratings are anchored by previous ratings. The average of the three ratings is the Baseline (BL). Once neurofeedback begins, the client is asked to make the same ratings at each session. Reduction in symptoms is calculated by averaging the ratings for each concern for the last three sessions. For example, the Average at 10 would be% change in rated concerns for sessions 8, 9 and 10 compared to the BL. For Average at 20 it would compare the BL with sessions 18, 19 and 20. Figure 1 shows our current data, updated daily to our website.

Certainly there are limitations with this approach to measuring change. It is subjective and susceptible to being affected by a desire to please the therapist by reporting improvement. The baseline is not a traditional baseline where ratings are taken over a span of time. Rather it is taken in one session and as noted earlier, the clients asked to give ratings for a “bad” week, a “good” week, and a “usual” week. This was done as clients who come for help are unlikely to be willing to come to sessions where there is no intervention simply to provide data. Finally, it only demonstrates that neurofeedback has the client reporting feeling better. For these reasons, the Results Tracking System (RTS), includes several other measures of impact. However, we should not minimize the power of this single measure. It is based on what the client is concerned about and uses the client’s ratings of whether they see improvement.

The results here appear strong with clients reporting a 37% reduction in the severity of their concerns after 20 sessions of neurofeedback. The shape of the reduction also suggests validity of the measure with more improvement occurring earlier in treatment. Note that neurofeedback is not a prescribed course of treatment and that the number of subjects decline as clients continue or finish therapy. The live data on the website shows the N’s for each data point.

### Impact on Behavior

**FIGURE 2:**
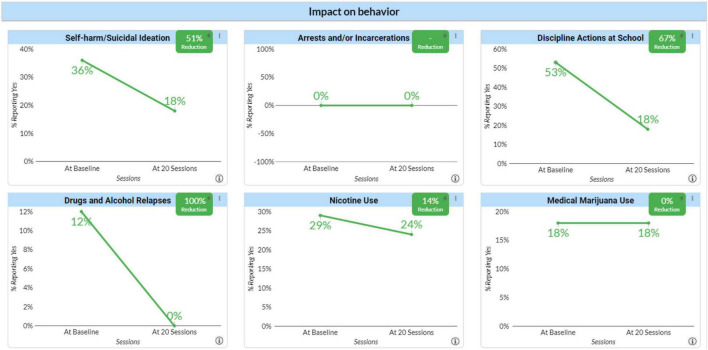
Impact of neurofeedback on behavior. Clients reported the occurrence of behaviors such as self-harm/suicide ideation, arrest and/or incarceration, discipline action at school, drug and alcohol relapses, nicotine use, and marijuana use before and after receiving neurofeedback therapy.

To assess the impact of neurofeedback on their behavior we selected six areas: Self-harm/Suicidal Ideation, Arrests/incarcerations, Disciplinary Actions at School, Drug or Alcohol Relapses, Nicotine Use and Medical Marijuana Use. We included medical marijuana rather than licit and illicit drug use from concern that clients might not report illicit use. For all these measures, each client or in the case of children, their parents were asked at baseline and again after 20 and 40 sessions whether the behavior had occurred either in the year prior to starting neurofeedback or in the time of the previous 20 sessions. Figure 2 shows the percentage of clients at baseline (BL) who reported such a problem and the percentage of just those clients who reported the problem to have continued since starting neurofeedback and the percentage change represented by the difference in the former and latter scores. The results are quite dramatic aside from Nicotine and Medical Marijuana use. Yet this relative lack of improvement may support the validity of the RTS as no agency that we are aware of made nicotine or marijuana use a focus of treatment. It also suggests that improvements do not simply reflect a desirability response bias.

### Impact on Cognition

**FIGURE 3:**
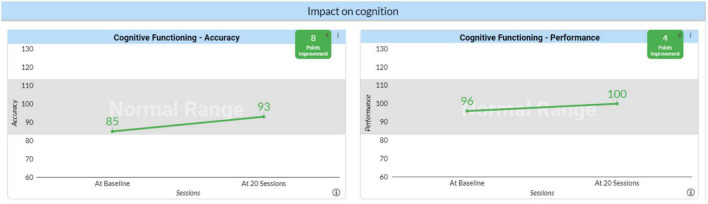
Impact of neurofeedback on cognition. Cognitive accuracy and performance outcomes before neurofeedback (baseline) and following 20 sessions of neurofeedback therapy.

To assess the impact of neurofeedback on client cognition, we used the QIK test, a stand-alone computerized visual Continuous Performance Test (CPT) developed for assessing attention and impulse control. A simple visual target or non-target is presented once every 2 s. During the 21-min test, the subject must quickly press a button for each target and not press for each non-target. The length and monotony of the tests makes it a good test of certain cognitive parameters. The QIK Performance Index reflects speed and consistency of response, which are continuous variables. The QIK Accuracy Index reflects sustained attention and impulse control, which involve discrete errors. Results are reported at BL and after 20 sessions. Testing is always preceded by a brief practice session and there is no learning effect from repeated administrations so changes should objectively represent change. The QIK Accuracy Index increased 8 points from a standard score of 85 to 93, a shift from the 16th to the 32nd percentile. The Performance Score increased 5 standard score points from 96 to 100, a shift from the 39th to the 50th percentile. Again, the results indicate that neurofeedback does improve cognitive performance (Figure 3).

### Impact on Healthcare Usage

**FIGURE 4:**
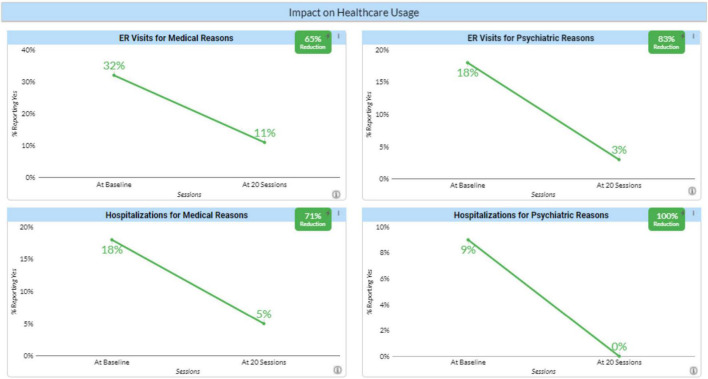
Impact of neurofeedback on healthcare usage. Percentages of clients reporting emergency room visits or hospitalizations for either medical or psychiatric reasons before and after 20 neurofeedback sessions.

A concern with any clinical innovation is whether it will result in an increased cost to our healthcare system. To implement neurofeedback requires equipment purchases and staff training. However, it is also known that the clients served by the agencies who are participating in the Neurofeedback Advocacy Project with high ACE scores have higher rates of healthcare utilization (Hargreaves et al., 2019). To assess its impact on healthcare utilization we asked whether the client had been to the ER for a medical or a psychiatric reason and whether they had been hospitalized for either a medical or psychiatric reason using the same time frames as the questions about behavior. Here the results for all four questions were very dramatic. As expected, the impact on ER visits and hospitalization was greater for psychiatric reasons than for medical reasons, but the impact on ER visits and hospitalizations for medical reasons was substantial (Figure 4). The relation between these improvements and the neurofeedback itself is unclear. Is neurofeedback so effective in treating not just psychiatric but also medical concerns? Does being in a therapy they find helpful lead clients to less costly interventions such as neurofeedback and counseling? These issues need to be investigated. But whatever the reason, they strongly suggest that neurofeedback is not a net cost to the system. Rather it represents an overall savings, even when regarded in a short time frame. That is to say, the return on investment (in the societal perspective) quickly turns positive.

### Coping With Stress, Side Effects, Medication Usage, Client Reviews, and Ratings

**FIGURE 5:**
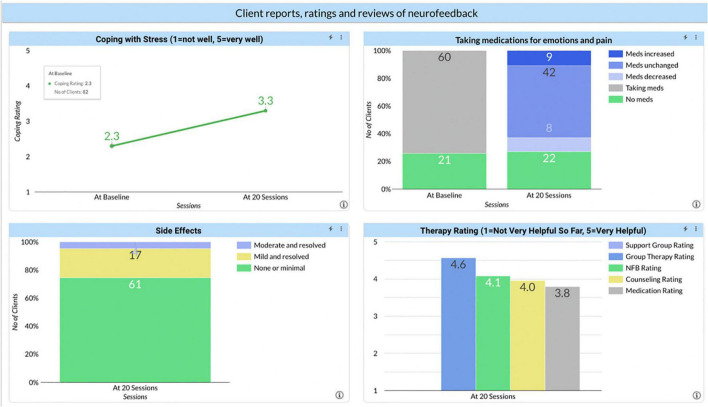
Client reports, ratings, and reviews of neurofeedback. Reported outcomes from clients at baseline and after 20 neurofeedback sessions for: coping with stress, taking medications for emotions and pain, side effects, and the helpfulness of therapy.

This is a group of disparate but important measures (Figure 5). The first, Coping with Stress was used because it was known that these clients already had a high level of stress in their lives. To get that measure, at baseline they were asked how many of the 10 psychosocial stressors in the DSM 5 they were experiencing. After getting that number, they were asked to rate how well they were coping on a scale of 1–5. After 20 and again after 40 sessions, they were asked about the number of stressors and their coping. The first chart shows an improvement of 1 point.

Asked about medications for emotions or pain, 61% of the clients reported they were taking something at baseline. Interestingly, that number did not change after 20 sessions; and among those who were taking medications, the same percentage increased as decreased their medication. The percentage of clients who were taking medication before treatment and no longer taking it after treatment was minimal. There could be several reasons. Possibly, neurofeedback had so little impact that a change in medication was not warranted. Just as likely, if not even more so, if a client is reporting doing better, the prescriber is likely to attribute the improvement to the medication and continue its use. As with nicotine and use of medical marijuana, most therapists did not make a reduction in prescribed medications a goal of therapy. Nor were most prescribers well informed, if even informed at all, about neurofeedback and how to titrate medication in that light.

We asked about adverse effects (typically called side effects). ILF Neurofeedback is a powerful intervention and part of the therapeutic process is getting the training frequency correct. When this is not the case, the client is likely to experience signs of either over or under-arousal or worsening of cerebral stability. Learning to identify these signs and make the appropriate adjustment is central to the training, but it is always possible that the training frequency is not optimal. Typically, if it is wrong and the client discontinues the therapy for reasons of adverse response, over time the brain will move back to its accustomed state, because the training effects will not yet have been consolidated. The subsidence of adverse responses will most likely occur over the course of a few days. What we do see is that while 25% of the clients reported either mild or moderate side effects during the treatment, all reported that those side effects had resolved. This supports the notion that ILF Neurofeedback is very safe, when conducted by experienced hands.

Asked to rate neurofeedback vs. other therapies they were receiving, neurofeedback was rated higher than counseling or medication, but lower than group therapy. The number of clients receiving group therapy is much lower than the other interventions, so it is unclear if this is an artifact of low N’s for group therapy or that this an under-utilized treatment. There is also some confusion because we view neurofeedback as a tool in psychotherapy, so a better comparison at some point would be psychotherapy alone vs. psychotherapy that included neurofeedback. We also asked clients to write a brief review of neurofeedback and posted those reviews to our website. Again, clients speak highly of it.

There are two other measures that are also dramatic. The first is the 2% No Show/Late Cancelation rate for clients compared with rates typically between 25 and 45% in agencies that serve difficult clients. Indeed, several agencies reported calls from clients to increase the frequency of visits or to call them if there was an opening in the schedule. Finally, we had therapists rate the termination status of clients. Choices included Finished or either Discontinued Not Related to the Treatment or Discontinued Related to the Treatment. The latter suggested dissatisfaction with the therapy while the Discontinued Not Related to the Treatment would include situations such as the client moving away. This last group is not included in the calculation of Premature Terminations. Again, the figure of 16% Premature Terminations is much lower than is commonly reported for any other therapy and suggests that clients both find benefit and enjoy the process.

There are obvious limitations to these data. As a type of Patient Reported Outcome Measure (PROM), the reduction in symptom severity is a subjective measure and formal reliability and validation studies have not been conducted. Similarly, the measures on changes in behavior and healthcare utilization have not been independently verified. Most importantly, none of these measures has been collected in any outcome comparison study. There are indications of validity. As discussed earlier, both nicotine and medical marijuana usage did not improve very much, suggesting that the data may not be skewed by a desire to please the therapist. Also, improvements were shown on an objective measure of cognitive performance.

## Conclusion and Future Directions

The Neurofeedback Advocacy Project itself continues to seek new agencies and affiliations with other organizations working to improve mental health and recently extended our work to European and Australian providers of neurofeedback. What started as a side project by the author has developed into a growing project with a Board of Directors, non-profit 501(c)(3) status, and a few paid employees. A current focus is developing a financially sustainable model for the project while not making costs a barrier to implementation of neurofeedback. Working with a Wicked problem means there really is no end point, no settled question.

Clearly there is a lot of work to be done. The clinical intake has been made central which both facilitates therapist learning and adherence to the treatment model. This, combined with the current session-by-session tracking of client progress further enhances quality assurance.

The software platform for the RTS was revised to allow it to hold huge amounts of data for “big data” analyses. For the first time we will have a constant feedback loop between client demographics, treatment protocols and clinical outcomes. This allows frequent updates to our products and procedures, something akin to what happens in nearly all industries except mental healthcare. This can lead to better training of clinicians and quality assurance as we monitor each clinician’s progress with each client and suggest where a clinician may need to revise their treatment plan.

The need is great, the challenges daunting. Adding ILF neurofeedback to clinicians’ therapeutic toolbox appears to improve the lives of the clients in significant ways. If, as our data suggests, neurofeedback can remediate disrupted neurodevelopment, the societal return will be extremely high. Recognizing that we are working with a Wicked problem is both humbling and exciting. No treatment can “solve” the problems affecting our current mental health care system. But the model we are following: implementing a novel but promising technology and using continuous, systemic monitoring of treatment and outcomes, holds the promise that we can affect significant improvements that matter.

## Data Availability Statement

The original contributions presented in the study are included in the article/Supplementary Material, further inquiries can be directed to the corresponding author.

## Author Contributions

MF designed and conducted the experiments, analyzed and interpreted the data, and prepared the manuscript.

## Conflict of Interest

The author declares that the research was conducted in the absence of any commercial or financial relationships that could be construed as a potential conflict of interest.

## Publisher’s Note

All claims expressed in this article are solely those of the authors and do not necessarily represent those of their affiliated organizations, or those of the publisher, the editors and the reviewers. Any product that may be evaluated in this article, or claim that may be made by its manufacturer, is not guaranteed or endorsed by the publisher.

## Footnotes

1
www.ResultsTrackingSystem.com

2
www.NeurofeedbackAdvocacyProject.com
