# Supplementary material for: Documenting the Impact of Infra Low Frequency Neurofeedback on Underserved Populations With Complex Clinical Presentations
Source: Front Hum Neurosci. 2022 May 26;16:921491. doi: 10.3389/fnhum.2022.921491 (PMC9198971; doi:10.3389/fnhum.2022.921491)
Supplement: Supplementary file 1 [file Data_Sheet_1.PDF]

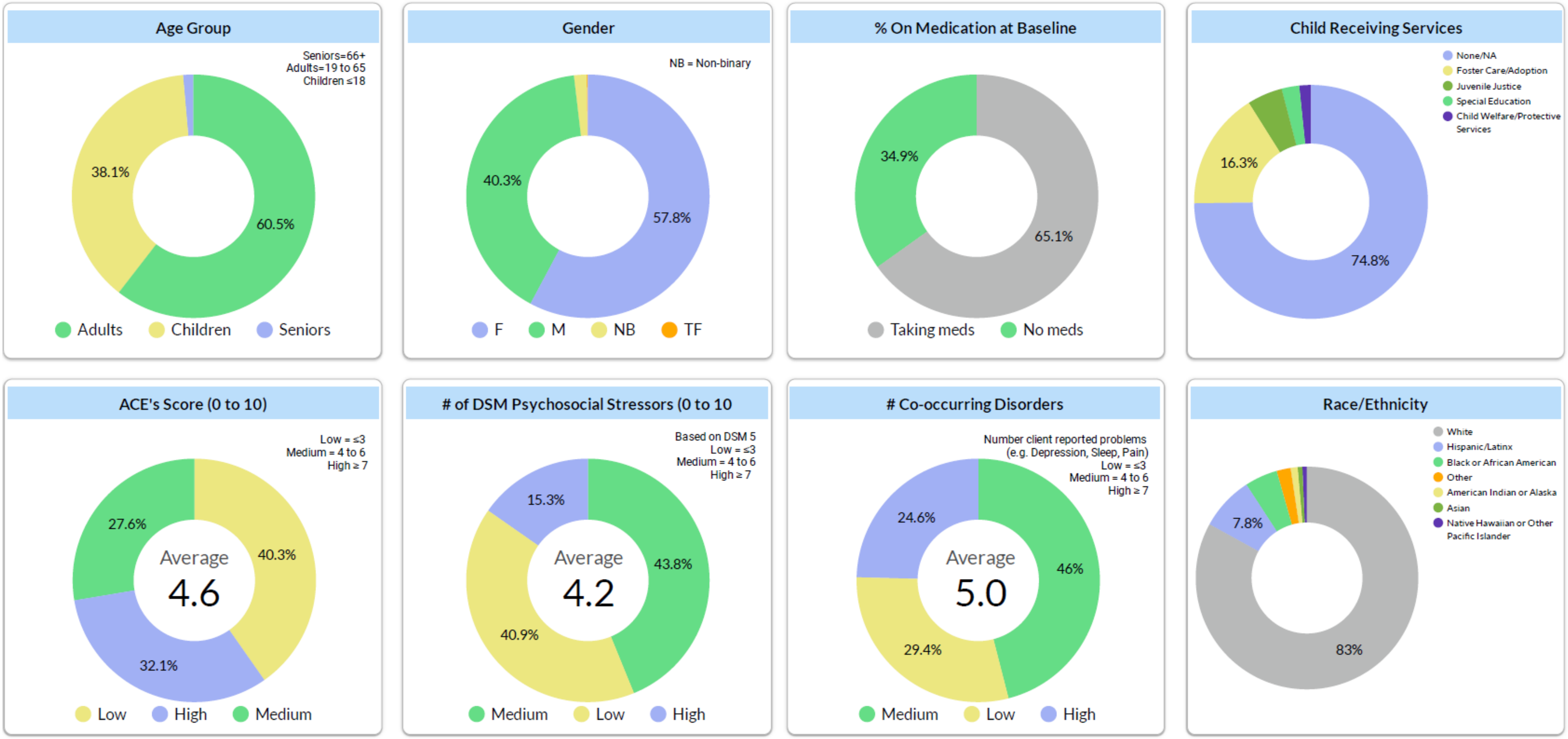

Supplementary Figure 1. Participant Demographics

Reported demographic measures for clients include age group, gender, percentage of clients taking medications at baseline, children receiving services (foster care, juvenile justice, special education, child welfare/protective services), ACE's scores, number of DSM psychosocial stressors, number of co-occurring disorders, and race/ethnicity.
